# Predictors of delayed treatment of Kawasaki disease in community and tertiary care hospitals

**DOI:** 10.1186/1546-0096-10-S1-A89

**Published:** 2012-07-13

**Authors:** Nadia Luca, Joyce CY Ching, Cedric Manlhiot, Brian W McCrindle, Rae SM Yeung

**Affiliations:** 1 Hospital for Sick Children, Toronto, ON, Canada

## Purpose

The aim of this study was to define a comprehensive profile of patients at risk for a delayed diagnosis of Kawasaki disease (KD) including non-patient related variables such as caregiver behavior and health care providers’ experience with KD.

## Methods

From 1995 to 2006, all hospitals and pediatric cardiologists in Ontario were contacted to identify all children diagnosed with KD. The following data were retrieved: demographics, day of week admitted, symptoms, clinical features, and treatment. Hospital KD caseload was defined as low (<20 cases/year) or high (≥20 cases/year). The only institution with a KD program was the Hospital for Sick Children in Toronto. The primary outcome was the number of days of fever prior to treatment with intravenous immunoglobulin (IVIg). Secondary outcome was the number of days between admission and treatment with IVIg. Data analysis was performed using multivariable linear and logistic regression models. The estimate* (est) reflects the change in the outcome (days) associated with a 1 unit increment (if continuous) or the presence (if binary) of the variable. The analysis was carried with and without data from the tertiary care centre.

## Results

2378 patients were included, 1472 (62%) of which were male. Median age was 3.2 years (range 0.05-22.0), and 73% were ≤4 years. Thirty percent of patients had <4 clinical features of KD. The median number of days of fever at diagnosis was 6 (range 0-30). Eight percent of patients had >10 days of fever at admission, and 13% were treated with IVIg at >10 days of fever.

Patients seen at lower KD caseload hospitals had fewer days of fever at admission (est 0.0031; *p*=0.005), but hospital volume did not impact number of days of fever at IVIg treatment (*p*=NS). Lower hospital volume was associated with a greater delay between admission and IVIg treatment (est 0.002; *p*=0.01). Thus, although patients presented to lower caseload hospitals earlier in their illness, treatment with IVIg did not occur until later. The most significant factor associated with delay in treatment was day of presentation. Children admitted on Sunday (est 0.80; *p*=0.04) or Monday (est 0.69; *p*=0.06) had more days of fever at time of treatment with IVIg, pointing to a parent/family/work-related factor. Exclusion of the hospital with a KD program gave even more significant results (Sun: est 1.21; *p*=0.004 and Mon: est 0.78; *p*=0.05). Admission on Sunday was also associated with greater delay between admission and treatment (est 0.30 *p*=0.04; excluding tertiary care centre: est 0.54; p=0.001), pointing to a potential health care provider-related factor.

**Table 1:** Clinical features associated with a greater number of days of fever at time of IVIg treatment and delay between admission and treatment with IVIg.

	Greater number of days of fever at IVIg		Greater delay between admission and IVIg	
Parameter	Estimate*	P value	Estimate*	P value

Older age	0.01	<0.001	0.002	0.04
Lower number KD criteria	0.34	0.002	NS	NS
Absence of conjuctivitis	0.98	0.001	0.29	0.01
Presence of arthritis	1.37	<0.001	NS	NS

IVIg = intravenous immunoglobulin; *see definition in methods section; KD = Kawasaki Disease; NS = non-significant.

## Conclusion

Non-patient related risk factors associated with delayed diagnosis and treatment of KD include admission to low-caseload hospitals and admission on Sunday. This emphasizes the need for interventions to target both parents and health care professionals, especially those in locations with low KD case volume and where resources may be limited on weekends.

## Disclosure

Nadia Luca: None; Joyce C.Y. Ching: None; Cedric Manlhiot: None; Brian W. McCrindle: None; Rae S.M. Yeung: None.

